# Optimizing Laparoscopic Adrenal Surgery: A Retrospective Study of 83 Patients at a Tertiary Cancer Center

**DOI:** 10.7759/cureus.89621

**Published:** 2025-08-08

**Authors:** Mohamed I Fahim, Fady Shafeik, El Sayed Ashraf Khalil, Abdelmaksoud M Ali, Rasha M Allam, Marina Asaad

**Affiliations:** 1 Surgical Oncology, National Cancer Institute, Cairo University, Cairo, EGY; 2 Oncosurgery, National Cancer Institute, Cairo, EGY; 3 Surgical Oncology, National Cancer Institute, Cairo, EGY; 4 Community and Family Medicine, National Cancer Institute, Cairo, EGY; 5 Paediatrics, Cairo University Hospital, Cairo, EGY

**Keywords:** adrenal tumor surgery, gauze retractor technique, laparoscopic adrenalectomy - adrenal tumor surgery - minimally invasive adrenalectomy - gauze retractor technique - port site modification, minimally invasive adrenalectomy, port site modification

## Abstract

Aim of the study

This study aims to report our institutional outcomes of laparoscopic adrenalectomy, to compare these with a cohort of patients undergoing open adrenalectomy, and to describe the feasibility and utility of technical modifications, including modified port positioning and the use of intra-abdominal gauze retractors.

Patients and methods

This retrospective cohort study included 83 patients with radiologically benign-appearing adrenal tumors who underwent adrenalectomy at the National Cancer Institute (NCI), Cairo University, Cairo, Egypt, between January 2014 and January 2020. Patient data were extracted from medical records and included demographics (age and gender), lesion characteristics (size, laterality, and functional status), surgical approach (laparoscopic or open), and results of endocrine investigations (serum metanephrines, urinary vanillylmandelic acid (VMA), aldosterone, cortisol, sodium, and potassium).

Results

Of the 83 patients, 57 (68.7%) underwent right adrenalectomy, and 26 (31.3%) had left adrenalectomy. Functioning adrenal lesions were identified in 32 patients (38.6%), including 31 pheochromocytomas and one cortisol-secreting tumor; the remaining 51 patients (61.4%) had non-functioning lesions. There was a statistically significant difference in pathological lesion size between patients undergoing open versus laparoscopic adrenalectomy (p = 0.014). No mortality was recorded in the open group; however, one postoperative death occurred in the laparoscopic cohort.

Conclusion

Laparoscopic adrenalectomy appears to be a feasible technique in selected patients with benign-appearing adrenal lesions, particularly for lesions up to 6 cm. Technical modifications, including modified port positioning and the use of gauze retractors, were subjectively found to facilitate operative exposure. However, due to the study’s retrospective design and limited sample size, no definitive conclusions can be drawn regarding comparative safety or effectiveness. Further prospective studies are needed to validate these findings.

## Introduction

Laparoscopic adrenalectomy has become the standard surgical approach for managing benign adrenal tumors, following its first description by Gagner et al. in 1992 [1]. Compared to traditional open adrenalectomy, the laparoscopic technique offers several notable advantages, including reduced postoperative pain, quicker recovery, shorter hospital stay, and improved cosmetic outcomes [1,2].

The anatomical location of the adrenal glands - deep within the retroperitoneal space - makes surgical access inherently challenging. Achieving sufficient exposure via open surgery often necessitates a large incision disproportionate to the gland's size. In contrast, a laparoscopic approach enables precise access with minimal disruption, making it a favorable option when oncological safety can be maintained [2,3].

This study presents our institutional experience with laparoscopic adrenalectomy, reporting institutional outcomes of laparoscopic adrenalectomy, comparing them with a cohort of open adrenalectomy cases, and describing the feasibility and utility of technical modifications, including modified port positioning and the use of intra-abdominal gauze retractors.

It also explores how tumor size influenced the choice of surgical approach and outcomes. Both elements are examined in the context of a real-world, evolving laparoscopic practice at a tertiary oncology center.

## Materials and methods

We conducted a retrospective observational cohort study at the National Cancer Institute (NCI), Cairo University, Cairo, Egypt. A total of 83 patients underwent adrenalectomy for radiologically benign‑appearing adrenal lesions between January 2014 and January 2020. Patient data were retrieved from hospital records and entered into a standardized database for subsequent analysis.

Patients were considered eligible if imaging demonstrated a benign‑appearing adrenal lesion, if they had a good performance status, and if baseline liver and kidney functions were preserved. Patients were excluded when pre‑operative imaging suggested malignant features such as irregular borders, infiltration of adjacent structures, or suspicious lymphadenopathy. Patients who were deemed unfit for surgery were also excluded.

**Figure 1:**
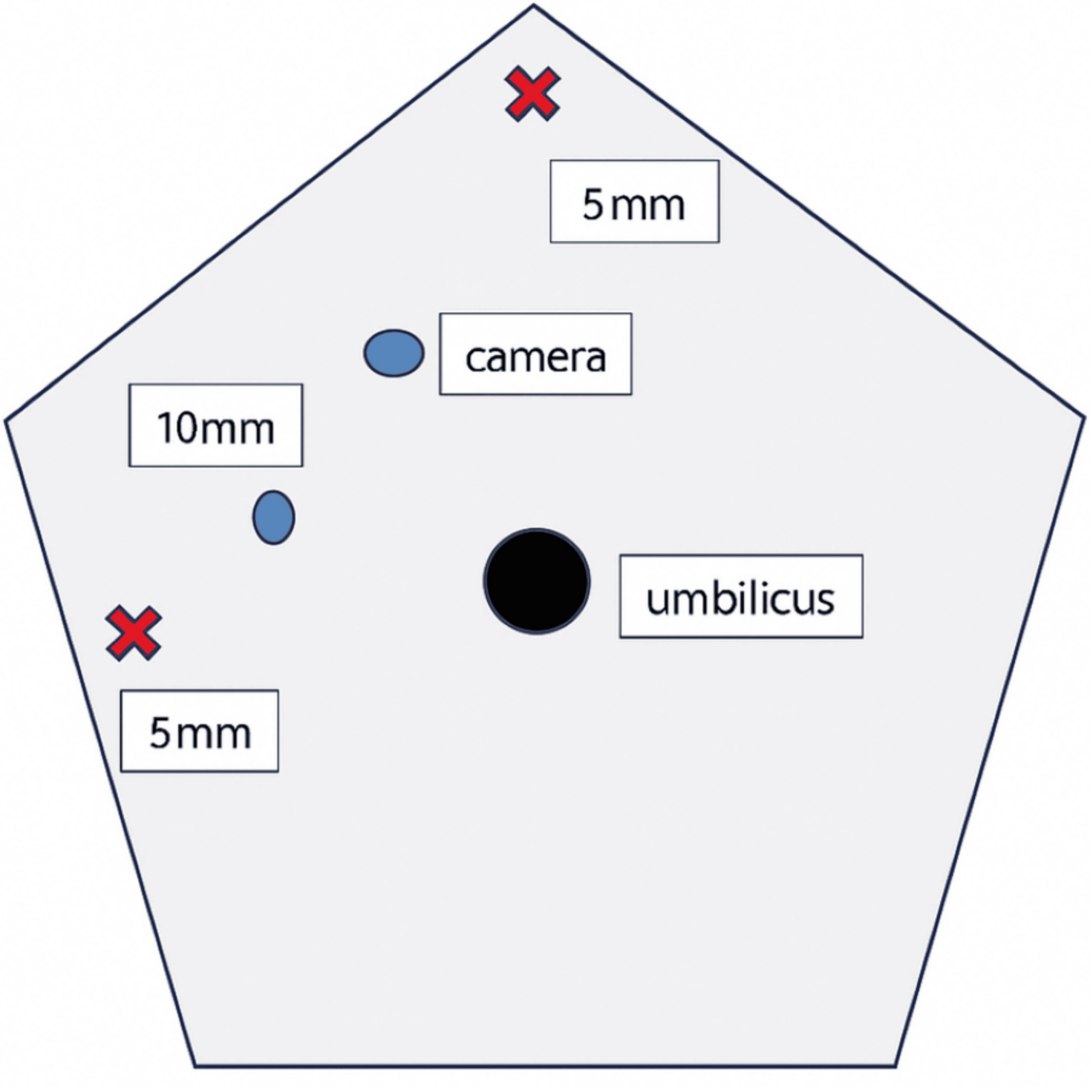
Port placement for right-sided laparoscopic adrenalectomy

**Figure 2:**
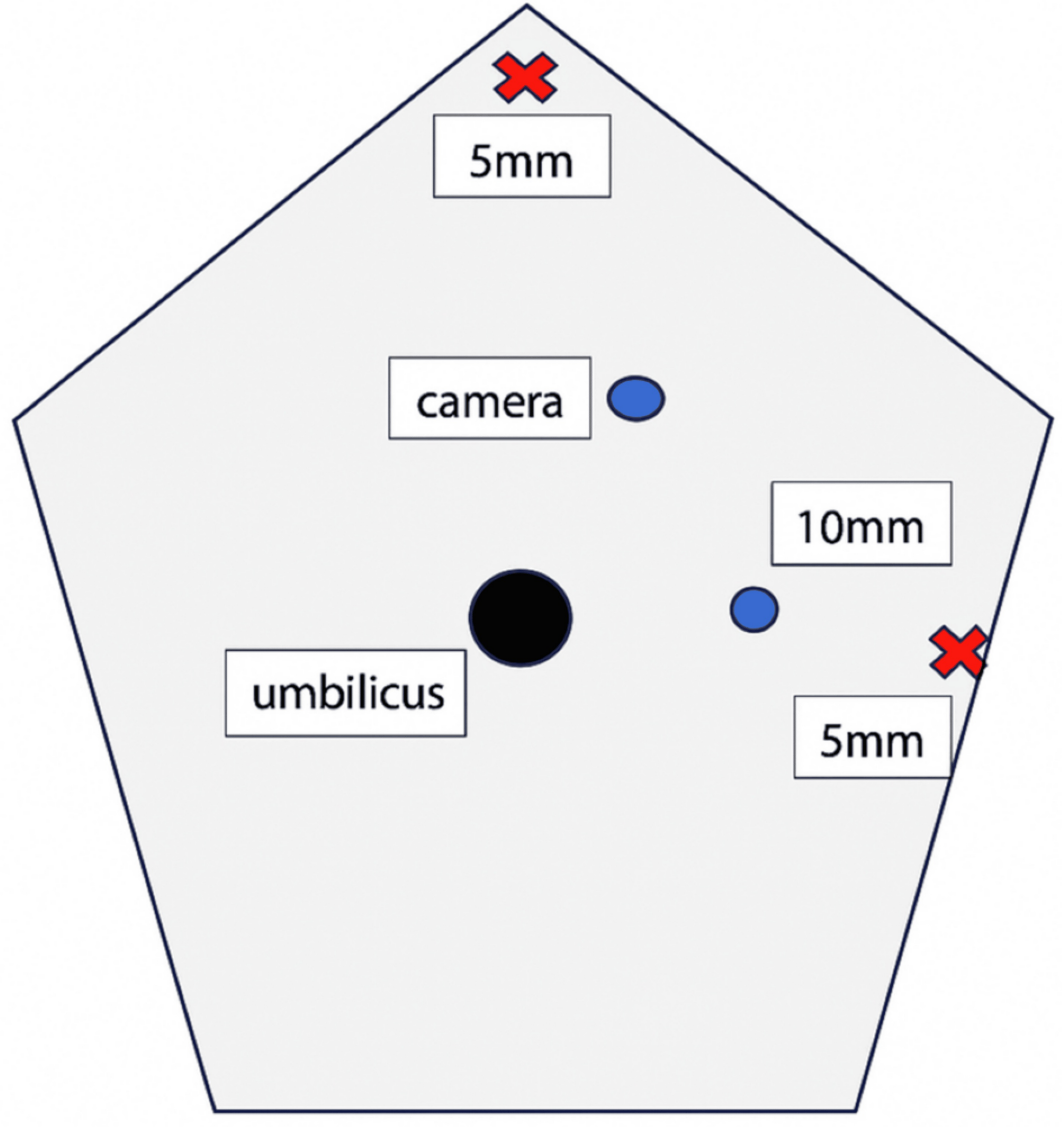
Port placement for left-sided laparoscopic adrenalectomy

**Figure 3:**
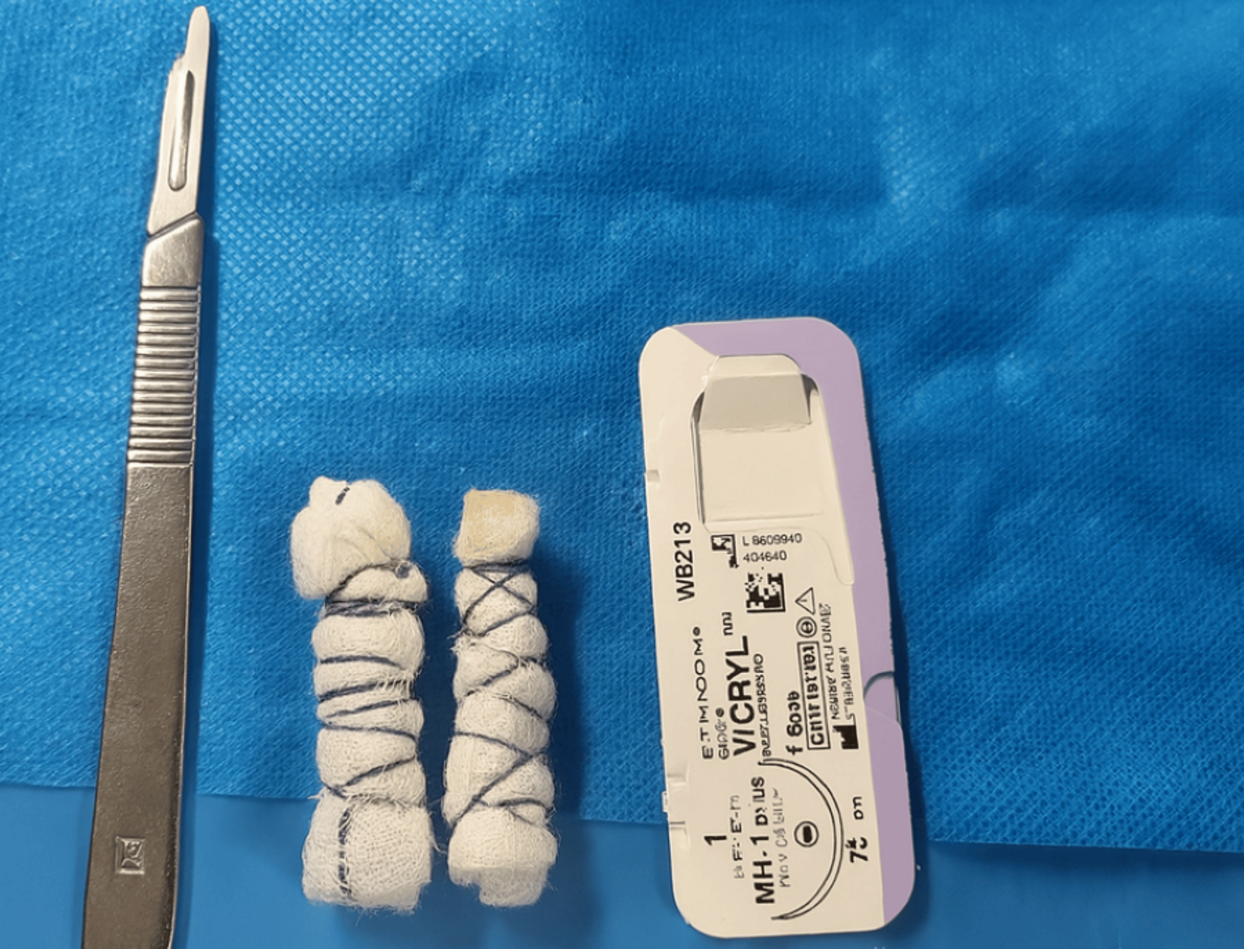
Intra-abdominal gauze retractors prepared for use

**Figure 4:**
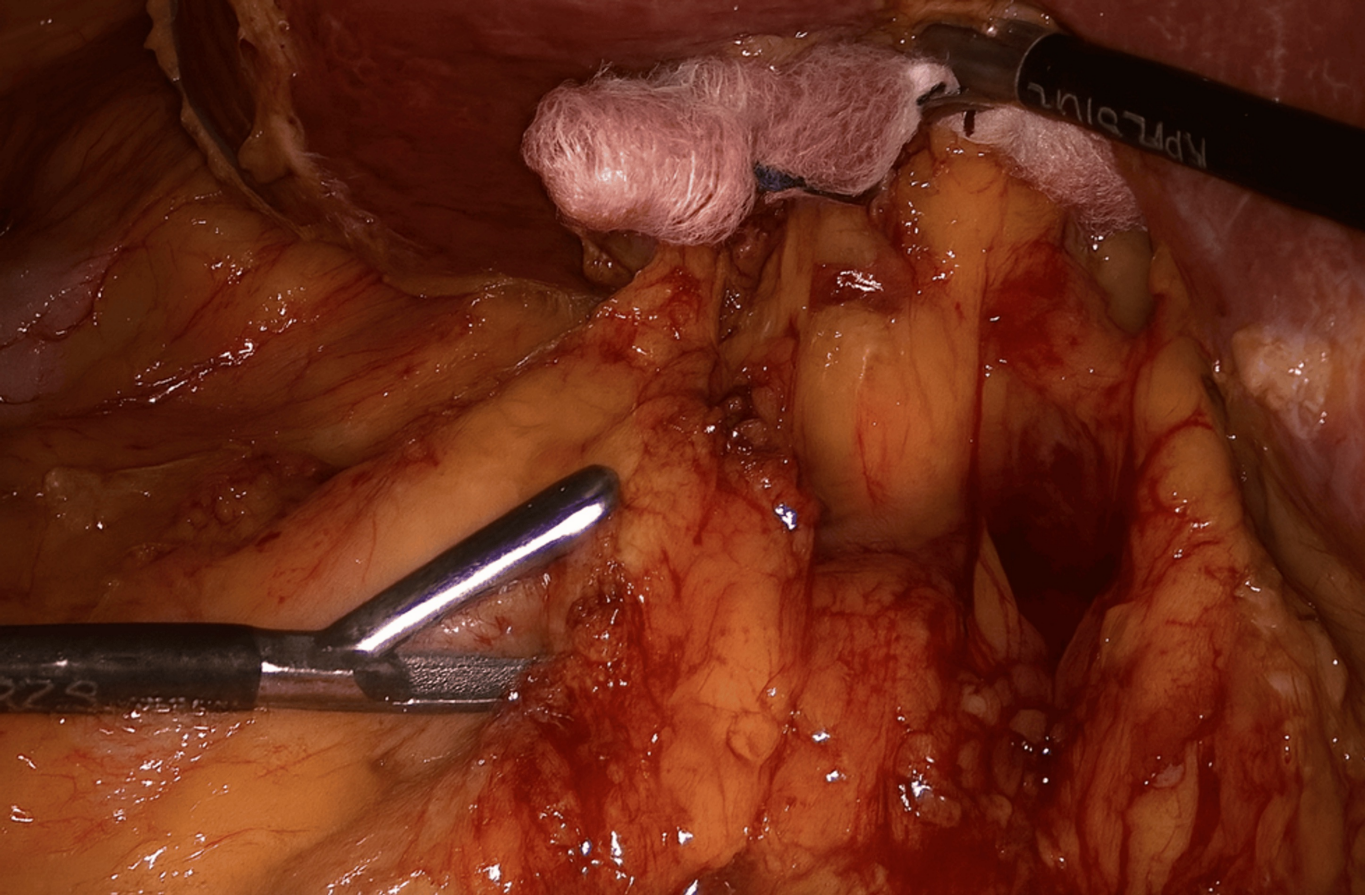
Application of the gauze retractor during right-sided adrenalectomy

**Figure 5:**
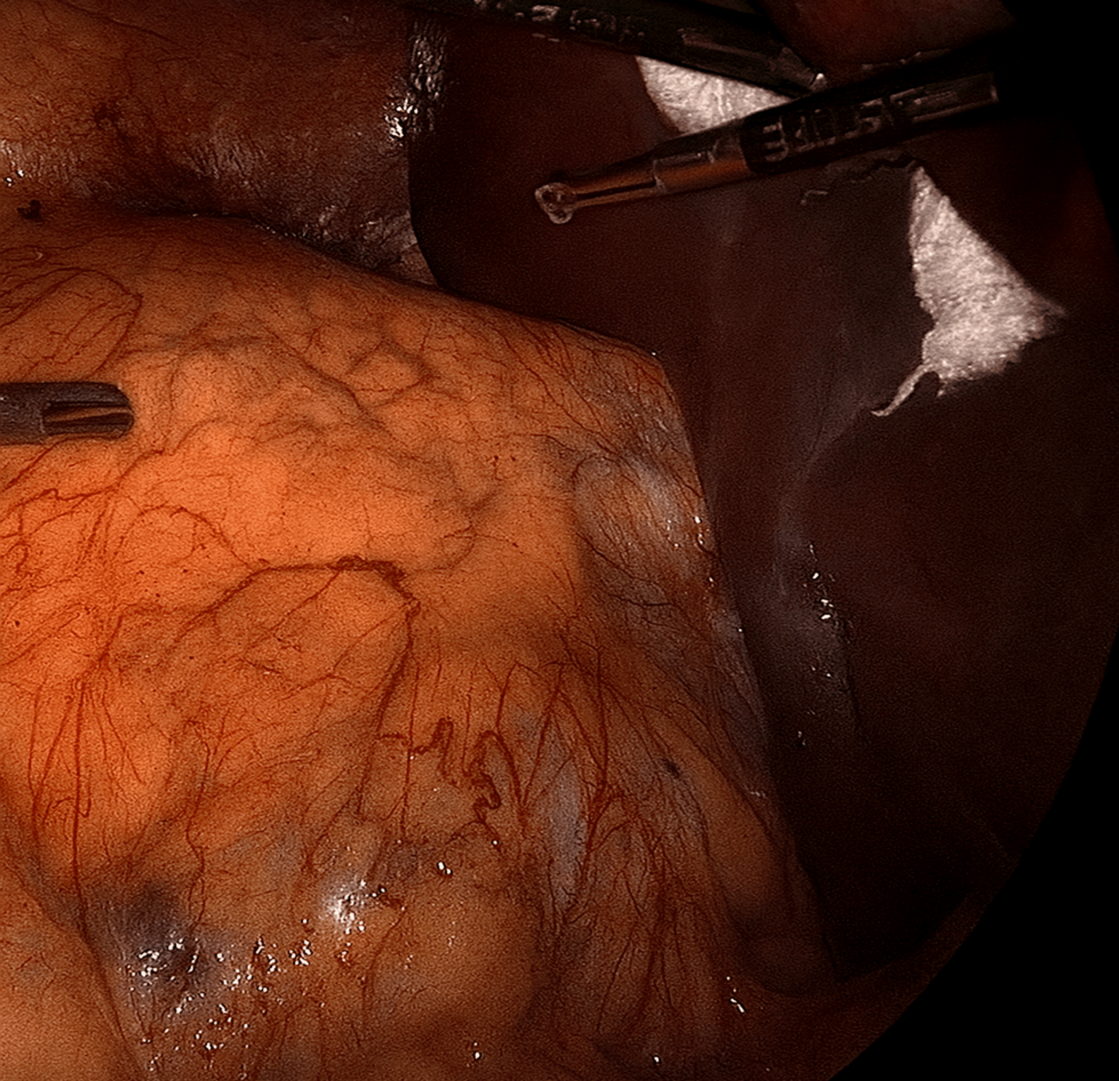
Alternative view showing the gauze retractor on the right side

**Figure 6:**
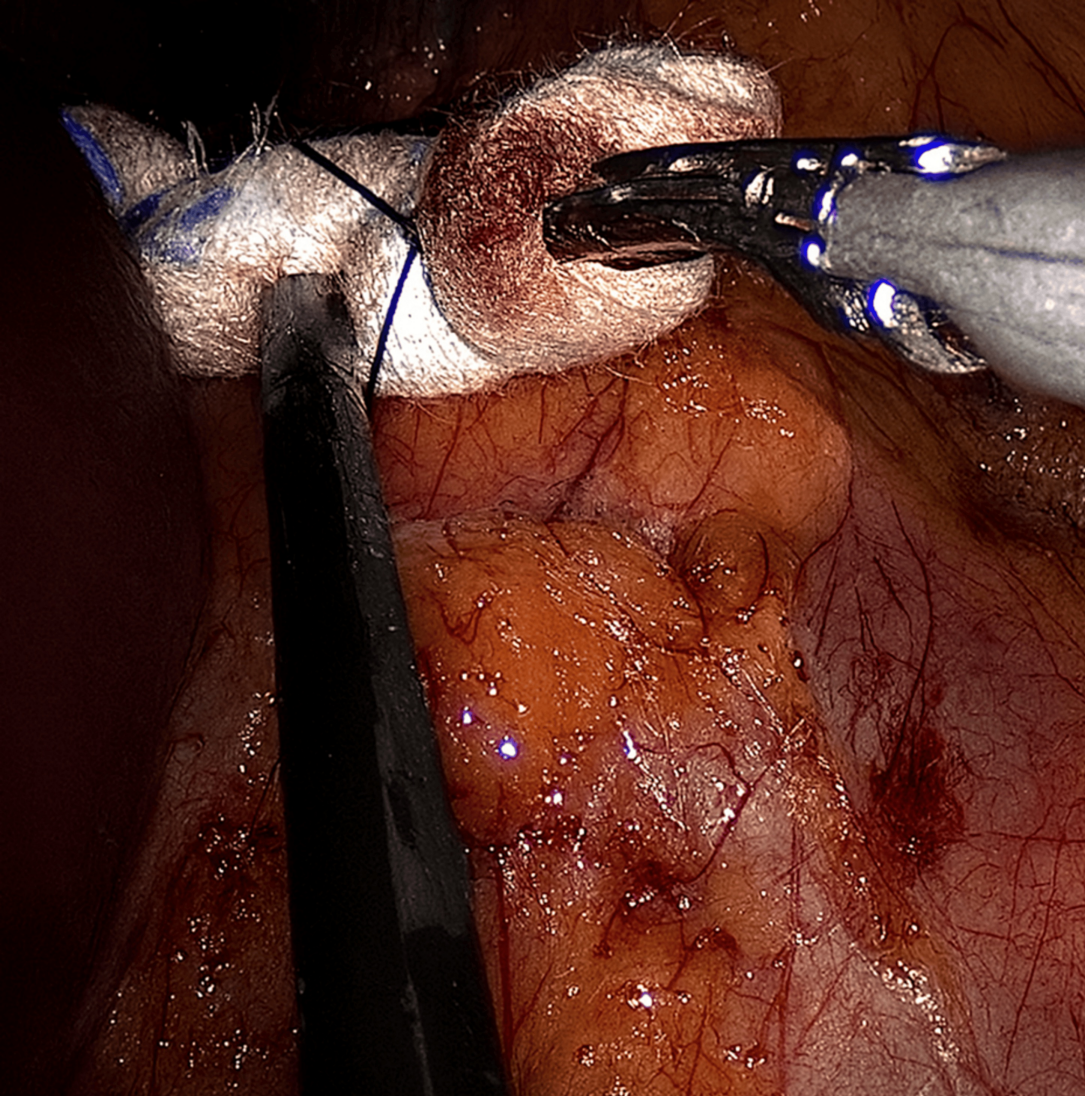
Use of the gauze retractor during left-sided adrenalectomy

**Figure 7:**
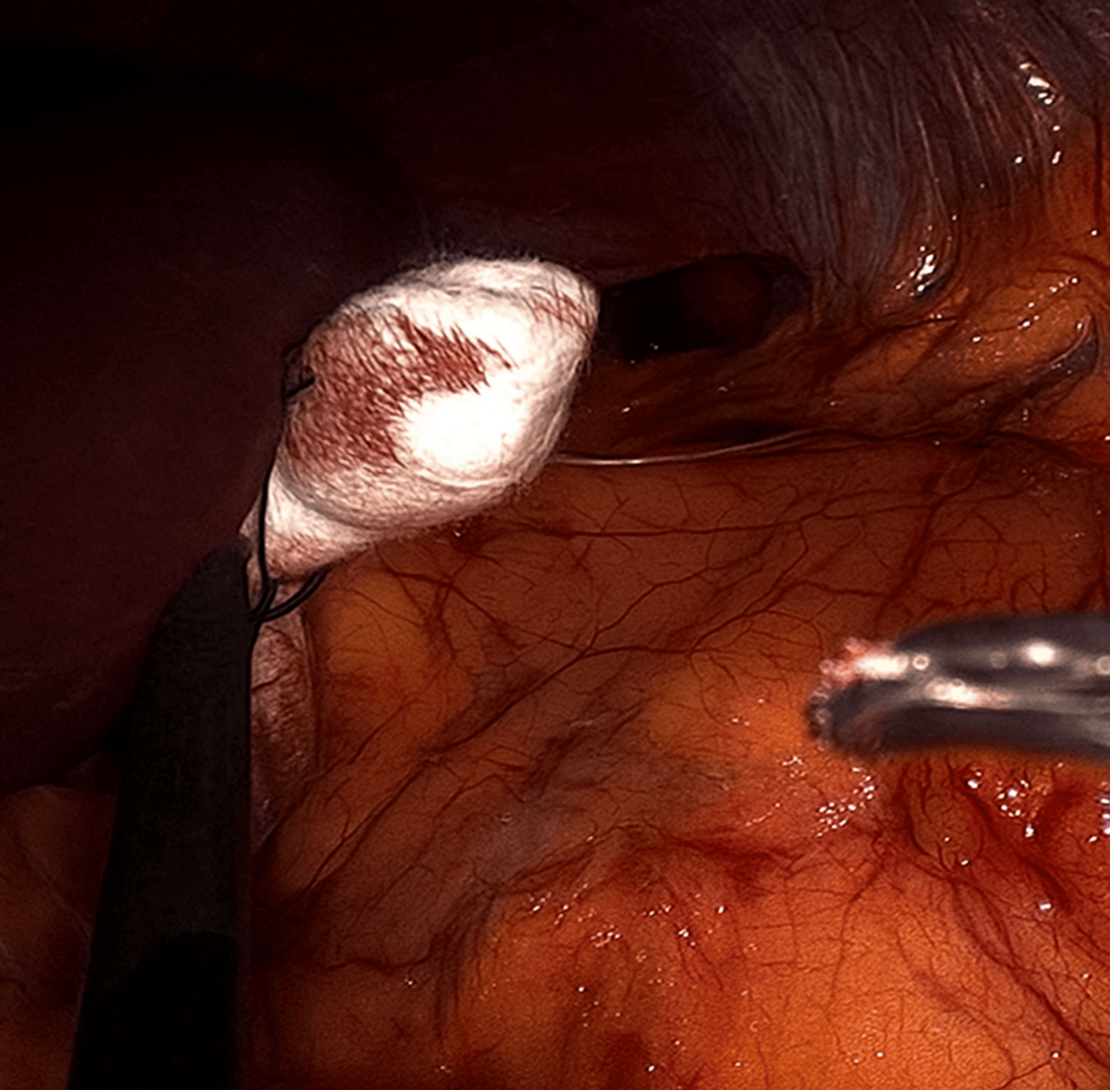
Additional view of the gauze retractor in the left-sided procedure

Among the 83 patients, 51 (61.4%) underwent open adrenalectomy, and 32 (38.6%) underwent laparoscopic adrenalectomy. All laparoscopic procedures were performed with the patient in a semi‑lateral position. Four ports were used: one 10‑mm camera port, one additional 10‑mm port, and two 5‑mm ports. For optimal retraction, a small piece of gauze was rolled and tied with 1‑0 Vicryl and introduced through the 10‑mm port to serve as an intra‑abdominal retractor. This method was adopted as a simple, flexible, and atraumatic alternative to rigid instruments, particularly useful in cases where deeper retroperitoneal access was required and advanced laparoscopic tools were not routinely available. It reflects the early phase of laparoscopic practice development within our unit. The port placements and retraction technique are illustrated in Figures 1-7.

The decision regarding surgical approach was individualized and made by the operating surgeon based on multiple factors, including tumor size and characteristics, patient comorbidities, prior abdominal surgery, and the surgeon’s laparoscopic expertise. As this study took place during the early phase of laparoscopic adoption in our unit, surgeon preference and case complexity played key roles in determining the approach.

The procedures were performed by senior consultant surgeons, including professors and lecturers in surgical oncology, with more than 10-20 years of operative experience at our institution.

Data collected for each patient included demographic details (age and sex), lesion characteristics (side and size), endocrine profile (serum metanephrines, urinary vanillyl mandelic acid (VMA), aldosterone, cortisol, sodium, and potassium), the chosen surgical approach (laparoscopic or open), and histopathological results.

Tumor size was assessed preoperatively using cross-sectional imaging and confirmed by pathological examination. Although it was considered during surgical planning, there was no fixed size cutoff; decisions were influenced by surgeon experience and unit expertise.

Postoperative follow‑up began one month after discharge and was continued at six‑month intervals. Follow‑up included imaging-computed tomography (CT) of the abdomen and pelvis and hormonal evaluations when indicated, particularly in patients with functioning adrenal lesions. The median follow‑up duration for the cohort was 30 months.

All patients provided written informed consent prior to inclusion in the study. The study protocol was reviewed and approved by the local institutional ethics committee.

Data were analyzed using Statistical Product and Service Solutions (SPSS, version 22; IBM SPSS Statistics for Windows, Armonk, NY). Quantitative variables were summarized as mean and range, and categorical variables were expressed as frequencies and percentages. Comparisons between open and laparoscopic groups for categorical variables were performed using the chi‑square test or Fisher’s exact test where appropriate. Continuous variables were compared using Student’s t‑test. A p‑value of less than 0.05 was considered statistically significant.

## Results

A total of 83 patients with adrenal tumors were included in this study and were followed throughout the designated period. The median age of the cohort was 47 years. Of these, 51 patients underwent open adrenalectomy, while 32 were managed laparoscopically. Detailed demographic and pathological characteristics are presented in Table 1. A statistically significant difference was observed in the pathological size of adrenal lesions between the open and laparoscopic groups (p = 0.014).

**Table 1:** Comparison between open and laparoscopic adrenalectomy groups regarding clinico-pathological characteristics Categorical variables were compared using the chi‑square test or Fisher’s exact test as appropriate. Test statistics and p‑values are shown.

Variables		Open (n=51)	Laparoscopic (n=32)	Test used and value	P value
Age (years)	<47	22 (43.1%)	18 (56.3%)	Chi-square = 1.354	0.245
	>=47	29 (56.9%)	14 (43.8%)		
Gender	Male	21 (41.2%)	8 (25.0%)	Chi-square = 2.263	0.132
	Female	30 (58.8%)	24 (75.0%)		
Laterality	Right	32 (62.7%)	25 (78.1%)	Chi-square = 2.162	0.141
	Left	19 (37.3%)	7 (21.9%)		
Size of lesion by radiology (cm)	<=6.5	22 (43.1%)	20 (62.5%)	Chi-square = 2.949	0.086
	>6.5	29 (56.9%)	12 (37.5%)		
Size of lesion by pathology (cm)	<=6	21 (41.2%)	22 (68.8%)	Chi-square = 5.988	0.014*
	>6	30 (58.8%)	10 (31.3%)		
Size of gland by pathology (cm)	<=5.5	23 (45.1%)	19 (59.4%)	Chi-square = 1.603	0.205
	>5.5	28 (54.9%)	13 (40.6%)		
Function	Functioning	19 (37.3%)	13 (40.6%)	Chi-square = 0.094	0.759
	Non-functioning	32 (62.7%)	19 (59.4%)		
Pathological diagnosis	Benign	29 (56.9%)	17 (53.1%)	Fischer’s exact = 0.335	0.919
	Malignant	2 (3.9%)	1 (3.1%)		
	Unpredictable behavior	20 (39.2%)	14 (43.8%)		
Pathological diagnosis	Benign	29 (56.9%)	17 (53.1%)	Chi-square = 0.111	0.739
	Malignant & unpredictable behavior	22 (43.1%)	15 (46.9%)		

Histopathological analysis revealed that 47 patients had benign adrenal lesions, including adenomas, ganglioneuromas, myelolipomas, cysts, and hematomas. A further 34 patients were diagnosed with borderline tumors, primarily pheochromocytomas, and three patients were found to have adrenocortical carcinoma based on postoperative pathology.

With respect to lesion function, 51 patients (61.4%) presented with non-functioning adrenal tumors, while the remaining 32 patients (38.6%) had functioning lesions. Among these, 31 were confirmed to have hormonally active pheochromocytomas, and one patient had a cortisol-secreting tumor.

In the laparoscopic group, two cases - both involving right-sided adrenalectomy - were converted to open procedures due to intraoperative bleeding. One of these patients experienced a fatal postoperative complication, resulting in mortality on the first day after surgery. In contrast, no postoperative deaths occurred among patients who underwent open adrenalectomy. All patients in both groups remained disease-free during follow-up, with no clinical or radiological evidence of recurrence or metastasis.

## Discussion

In this study, the majority of adrenal tumors were non-functioning, comprising 61.4% (51 of 83 cases), while the remaining 38.6% (32 patients) had functioning lesions. These findings are consistent with previously reported literature, which has shown that non-functioning adrenal tumors represent the most common clinical presentation [4,5].

Tumor size plays a critical role in determining the surgical approach. Several authors have advocated for a maximum lesion size of 6 cm for laparoscopic adrenalectomy, citing increased technical challenges and a higher probability of malignancy with larger tumors [6,7]. However, other studies have reported successful laparoscopic resection in select cases with lesions up to 8 cm in diameter, particularly when preoperative imaging and hormonal evaluation do not suggest malignancy [8,9].

The choice of surgical approach in this study was not based on a strict size threshold. Although a 6 cm cutoff is often used in the literature to guide laparoscopic feasibility, decisions were influenced by surgeon experience, tumor characteristics, and intraoperative conditions. During the study period, laparoscopic adrenalectomy was still being adopted in our unit, and as a tertiary oncology center, open surgery remained the default in many cases - including some with lesions under 6 cm.

In our cohort, one-third of patients with lesions larger than 6 cm (10 out of 30) were managed laparoscopically, while the rest underwent open surgery. The pathological lesion size was significantly different between groups (p = 0.014), supporting the cautious selection of laparoscopic cases based on tumor characteristics, size, and surgical expertise.

According to the European Society of Endocrinology Clinical Practice Guidelines, features such as tumor size exceeding 4 cm, irregular margins, heterogeneous appearance, and multiple hormone secretion raise suspicion for adrenocortical carcinoma and warrant consideration for open resection [10]. Similarly, the 2022 NCCN Guidelines for Neuroendocrine and Adrenal Tumors recommend open adrenalectomy in cases where imaging or biochemical profiles suggest malignancy.

Recent advancements in minimally invasive adrenal surgery are rapidly evolving. Robotic adrenalectomy has emerged as a promising alternative to conventional laparoscopy, offering enhanced dexterity, three-dimensional visualization, and improved ergonomics. Comparative studies and meta-analyses have demonstrated that robotic adrenalectomy may lead to shorter operative times, reduced blood loss, and similar oncological outcomes in appropriately selected cases [11]. In addition, technologies such as 3D laparoscopy, high-energy sealing devices, and intraoperative fluorescence imaging have been introduced to improve dissection accuracy and reduce complication rates [12]. The integration of Enhanced Recovery After Surgery (ERAS) protocols has further contributed to shorter hospital stays and optimized perioperative outcomes in adrenal surgery [13].

In addition, multi-institutional series have confirmed the safety and reproducibility of laparoscopic adrenalectomy across large patient cohorts, further validating its role as a standard approach for benign adrenal disease [14]. Moreover, recent prospective data highlight that optimized perioperative pathways, including individualized anesthesia protocols and minimally invasive techniques, continue to reduce morbidity and improve long-term functional outcomes after adrenal surgery [15].

In our experience, the use of gauze retractors appeared to improve operative exposure during laparoscopic dissection, particularly in cases requiring deep retroperitoneal access. Similarly, the modified port configuration was subjectively found to provide comfortable ergonomics for the surgical team. However, as no objective measures (e.g., operative time, blood loss, or standardized surgeon feedback) were used to quantify these aspects, these observations should be interpreted as subjective impressions rather than validated findings.

This study has several limitations. Its retrospective design introduces potential for selection bias and unmeasured confounding. The relatively small sample size, particularly when divided between laparoscopic and open groups, limits the statistical power of between-group comparisons. As a single-institution experience, the results may not be fully generalizable to other surgical teams or populations. Furthermore, the significant difference in pathological tumor size between the groups suggests that selection bias may have favored laparoscopic resection for smaller lesions. These factors should be considered when interpreting our findings, and further prospective multicenter studies are recommended.

While this study describes the feasibility of gauze retraction in laparoscopic adrenalectomy, it was not designed to compare outcomes between cases with and without this technique. Future prospective studies could further assess its relative benefits and clinical impact.

## Conclusions

Laparoscopic adrenalectomy appears to be a feasible technique in selected patients with benign-appearing adrenal lesions, particularly for lesions up to 6 cm. Technical modifications, such as modified port positioning and the use of gauze retractors, were subjectively found to enhance operative exposure and facilitate dissection. However, due to the study's retrospective design, selection bias, and small sample size, no definitive conclusions regarding comparative safety or superiority over open surgery can be drawn. Further prospective studies are warranted to validate these findings.
